# Aging, inflammaging and immunosenescence as risk factors of severe COVID-19

**DOI:** 10.1186/s12979-022-00309-5

**Published:** 2022-11-11

**Authors:** Anteneh Mehari Tizazu, Hylemariam Mihiretie Mengist, Gebreselassie Demeke

**Affiliations:** 1 Department of Microbiology, Parasitology and Immunology, School of Medicine, St. Paul’s Hospital Millennium Medical College, Addis Ababa, Ethiopia; 2 Department of Medical Laboratory Science, College of Medicine and Health Science, Debre Markos University, Debre Markos, Ethiopia

**Keywords:** Aging, Inflammaging, Immunosenescence, COVID-19, SARS-CoV-2

## Abstract

Coronavirus disease 2019 (COVID-19) is a respiratory infectious disease caused by the novel severe acute respiratory syndrome coronavirus 2 (SARS-CoV-2). COVID-19 is characterized by having a heterogeneous disease course, ranging from asymptomatic and mild symptoms to more severe and critical cases. In most cases the severity of COVID-19 is related to host factors, especially deregulation of the immune response in patients. Even if COVID-19 indiscriminately affects individuals of different age group, ethnicity and economic status; most severe cases and disproportional mortality occur in elderly individuals. This point out that aging is one risk factor for unfavourable clinical outcomes among COVID-19 patients. The biology of aging is a complex process; Aging can alter the structure and function of cells, tissues, and organs resulting in impaired response to stress. Alongside with other systems, the immune system is also affected with the aging process. Immunosenescence is an age associated change in the immune system that affects the overall response to immunological challenges in the elderly. Similarly, apart from the normal inflammatory process, aging is associated with a low grade, sterile, chronic inflammation which is termed as inflammaging. We hypothesized that inflammaging and immunosenescence could play an important role in SARS-CoV-2 pathogenesis and poor recovery from COVID-19 in elderly individuals. This review summarizes the changes in the immune system with age and how these changes play part in the pathogenesis of SARS-CoV-2 and clinical outcome of COVID-19 which could add to the understanding of age associated targeted immunotherapy in the elderly.

## Introduction

After it was first reported in Wuhan, China in December 2019, the novel severe acute respiratory syndrome coronavirus 2 (SARS-CoV-2), which is the causative agent of coronavirus disease 2019 (COVID-19) has become the worst pandemic of the twenty-first century. As of July 11, 2021, 183 million cases and almost 4 million deaths due to COVID-19 had been reported worldwide [1]. SARS-CoV-2 indiscriminately infects people regardless of age, sex, race or economic status, but old age by itself is one risk factor for developing a more severe clinical manifestation associated with COVID-19. Alongside aging, the presence of comorbidities like diabetes, lung disease, hypertension, and obesity lead to disease complications like severe pneumonia and acute respiratory distress syndrome (ARDS) [2, 3].

Coronaviruses were first identified in the early 1960s, characterized by having an envelope and a positive-sense single-stranded RNA viral genome [4]. Most strains of the human coronavirus (HCoV) cause a mild upper respiratory infection in immunocompetent hosts, but virulent forms of HCoV can cause serious life-threatening disease. Before the occurrence of the current SARS-CoV-2, the world has experienced two virulent forms of human coronaviruses. The first one was the severe acute respiratory syndrome coronavirus (SARS-CoV) which lasted between 2002 to 2003, and was first identified in Guangdong, China resulting 8000 cases worldwide. The second pathogenic coronavirus was the Middle East respiratory syndrome coronavirus (MERS-CoV) that occurred in 2012 which affected mainly the middle east with a total of 2000 cases reported [5, 6].

Deregulation of the immune system associated with age could have an important role in the pathogenesis of COVID-19 leading to serious clinical outcomes. The pathophysiology mechanisms of sever lung damage which is more common in elderly COVID-19 subjects could be associated with inflamm-aging”, mild-grade inflammation, and “cytokine cascade (“cytokine storm”) [7]. Aging is accompanied with different changes in the body, one of the changes occur in the immune system. Both the innate and adaptive immune systems show a different trajectory with age where such changes could be important factors for the severe clinical manifestation and disproportional higher mortality rate among elderly COVID-19 patients compared to children and young adults [2, 8].

For an effective defense against pathogens including viral infection, the body uses a coordinated mechanism of the immune system involving both the innate and adaptive immunity as per needed. In this review, we hypothesize age associated low-grade chronic inflammation (inflammaging) and age associated changes of the immune system (immunosenescence) observed in elderly individuals plays significant role in the pathogenesis and clinical outcome of COVID-19. Better understanding of these changes could be of a great importance in the development and response assessment of both therapeutics and COVID-19 vaccines. Here, we discussed major immunological changes associated with aging, how aging affects the pathogenesis of SARS COV-2 focusing on the dysregulation of the immune system with age and how it further complicates the clinical outcome of COVID-19 in elderly individuals.

### SARS-CoV-2 pathogenesis and epidemiology: implications for older adults

The first cases of COVID-19 patients were admitted by having unusual serious viral pneumonia of unknown origin. The source of the virus was back tracked and was linked to the wet animal wholesale and seafood market in Wuhan, Hubei Province, China. The viral aetiology agent was later identified as novel *coronavirus (*2019-nCov) (latter named as SARS-CoV-2). The whole genome sequence of SARS-CoV-2 showed that it shares 79.6% sequence similarity with SARS-CoV-1 while having 96% sequence identity with bat coronavirus [9–12].

SARS-CoV-2 use a spike (S) glycoprotein present on the virus’s envelope to attach itself to cell surface of the respiratory tract expressing the angiotensin-converting enzyme 2 (ACE2) receptor and S protein priming by the host cell transmembrane serine protease 2 (TMPRSS2). Besides the respiratory tract, ACE2 is expressed on the alveolar cells of the lung, myocytes of the cardiac, vascular endothelium and other cells of different tissues. After attachment, it can enter into the cell and replicate inside the cell causing disease [13, 14]. Most immune cells including CD4+, CD8+, B cells, Tregs, NK cells, Th17, NKT, monocytes, dendritic cells, and granulocytes express minimal to no level of ACE2. The presence of infected immune cells in sever COVID-19 cases could indicate the presence of a different receptor or other mechanism the virus use to infect these cells [15].

Individuals infected with SARS-CoV-2 show a wide range of heterogeneous clinical manifestations ranging from asymptomatic case to severe disease that can result death. Respiratory symptoms including cough, sneezing, and shortness of breath accompanying with high fever are the most common clinical manifestations. Other less common clinical symptoms includes diarrhea, nausea, vomiting and dysfunction of the vascular endothelial cells resulting in abnormal coagulopathy leading to thromboembolism and stroke [16–18]. The complex interaction of SARS-CoV2 with endothelial cells and pnemocytes result in dyregulation of the inflammatory and haemostatic system leads to coagulopathy. Some of COVID-19 patients are unable to control the virus resulting in apoptosis of pneumocytes and endothelial cells which aggravates inflammation. Severe inflammatory process leads to imbalance between procoagulant and anticoagulant homeostatic pathways resulting in coagulopathy in these patients [19]. Patients with severe COVID-19 coagulopathy tend to have a high level of proinflammatory markers, high D-dimer, prolonged prothrombin time, and reduced platelet counts [20, 21].

The median incubation period for developing the common symptoms of COVID-19 is  5.2 days [22]. The median day from the development of symptoms to the occurrence of death is 14 days and this period is shorter for elderly patients above the age of 70 (median number of days is 11.5). Besides developing the symptoms early, more death cases occur in elderly patients which could be linked to the uncontrolled viral progression due to weak immune system in this group [23]. The severity and the clinical outcome of COVID-19 differ between young and old patients, where elderly show a more severe clinical manifestation of the disease. One explanation is the difference in the pathogenesis of SARS-CoV-2 in young and old individuals [24]. Transmission of SARS-CoV-2 involves the deposition of viral infected respiratory droplets on conjunctival, oral, nasal, and mucosal membranes. The host cell receptors are expressed in the target cell making it susceptible for viral entry to the host. SARS-CoV-2 has a higher affinity to the ACE 2 receptor on the host cells which enable it to have higher transmission rate as compared to SARS-CoV-1 [25].

Poor prognosis were associated with increase in angiotensin 1–10 and a decrease in angiotensin 1–9 (processed by ACE2) among COVID-19 patients admitted in intensive care unit suffering with ARDS. This could suggest that a decreased activity of ACE2 in sever COVID-19 patients [26]. Multi-organ injury in COVID-19 patients were also linked with deregulation of ACE2 [27]. Functional and structural changes in the respiratory system occur with age. Older adults have a lower respiratory muscle strength which lowers the coughing reflex resulting in lower airway clearance of foreign material. Lower cough reflex associate with increased incident of pneumonia in elderly individuals [28]. These Physiological and anatomical changes of the respiratory tract in the elderly could predispose them for a more severe COVID-19 outcome.

ACE-2 is present on cells of different organs and used as receptor and facilitates the entrance of SARS-CoV-2 into the cell. Studies have variable results on the expression level of ACE2, where some studies reported an increased in ACE2 level with age [29]. which is in contrary to other studies reporting a decreased level of ACE2 with age [30, 31]. Apart from serving as a receptor, ACE2 has an anti-inflammatory role. ACE2 is part of the renin-angiotensin-aldosterone-system (RAAS) signalling pathway which plays a crucial role in converting the proinflammatory molecules angiotensin 2 to anti-inflammatory molecule angiotensin 1–7. The observed severe clinical outcome of COVID-19 in patients with cardiovascular disease and diabetes could be linked with the decreased expression of ACE2, that make them vulnerable to angiotensin 2 proinflammatory pathway [32]. Whereas others showed no difference in the expression level of ACE2 between young and old individuals [33].

In general, ongoing epidemiological data indicate the death rate due to COVID-19 is much higher in older individual and individuals with comorbidities compared to young once [34]. Age associated physiological changes in the respiratory tract like ciliary dysfunction makes old individuals unable to easily remove infectious agents and gives the pathogen time to enter into the cell and cause disease. The decrease in the ACE2 level and the associated increase in the proinflammatory environment could also be another reason for the observed severe clinical manifestation of COVID-19 in the elderly. This hypothesis is aligned with the role of ACE2 in protecting acute lung injury [35]. Elucidating the pathogenesis of SARS-CoV-2 and the difference in the expression level of ACE2 between young and old individuals should be further investigated. Factors like presence of comorbidity, gender, and genetic makeup should also be considered when assessing the role of ACE2 and age associated pathogenesis of COVID-19.

### Inflammaging in elderly individuals

The complex cellular and molecular event with concomitant immunological and physiological events associated with inflammatory process makes simplistic definition of inflammation challenging [36]. Nevertheless inflammation can be defined as a response to exogenous and endogenous stimuli generated from pathogens, traumatic, ischemic, physical, chemical or other challenges [37]. The inflammatory response differs depending on the tissue and organ where the stimuli generated. Different cytokines and chemiokines are released once the stimuli are sensed by cells like lymphocytes, fibroblasts and epithelial cells [36]. The role of inflammation is to help with clearance and repair tissue damage by recruiting the corresponding immune cells and molecules. The resolution of inflammation aims at restoring functions and structure of the tissue when the aggression is controlled. The movement of leukocyte and different proteins from circulation to the site of damage or infection is mediated by different players including vasoactive amine, cytokines, and chemokines. An increased blood flow, increased permeability of the blood vessels, increased secretion of chemokines, and increased expression of adhesion molecules facilitate leukocytes and proteins to pass through the blood vessels and move to the extracellular space around the damaged tissue. This movement results in accumulation of neutrophils at the area of damage which are then replaced by recruited monocytes that may differentiate into macrophages. The macrophages phagocytize and remove the debris which stops the damage signal and restores tissue function [38]. Resolution of inflammation is a highly regulated process involving anti-inflammatory molecules and lipid mediators like transforming growth factor-β1 (TGF-β1) and lipoxins respectively [39]. However, if the process of inflammation is not well controlled, it may result in chronic inflammation which causes malfunction of the tissue. For instance, rheumatoid arthritis results from the accumulation of inflammatory cells in the synovial joint which leads to chronic joint damage [40].

Apart from the normal inflammatory process, aging is associated with a low grade, sterile, chronic inflammation which is termed as inflammaging [41]. One of the common inflammatory biomarkers that have been frequently reported to increase with age is IL-6.. It is involved in acute phase inflammatory response, like it induce hepatic production of C-reactive protein (CRP) [42].

Beside that IL- 6 has been linked to age related pathologies such as autoimmune diseases and cancer [43]. It has been shown that, compared to young individuals, the level of IL-6 was higher in healthy old individuals without any clinical disease [44, 45]. Another important inflammatory biomarker that has been reported to increase with age is tumor necrosis factor alpha (TNFα). It is an important pro-inflammatory mediator involved in combating infection, but elevated circulating levels have a deleterious effect. Beyond increased levels in aging per se, TNFα has been associated with different age-related diseases [46].

The NLRP3 (NOD-, LRR- and pyrin domain-containing protein 3) is an intracellular pattern recognition receptor that is activated by a wide range of endogenous danger signals, *pathogen*-associated molecular *patterns* (PAMPs) and exogenous stimuli, resulting in NLRP3 inflammasome. The formation and activation of NLRP3 inflammasome elicits caspase 1-dependent release of the pro-inflammatory cytokines IL-1β and IL-18 [47]. NLRP3 inflammasome has been linked with age-related pathologies like metabolic disorders [48]. The presence of inflammaging in old individuals prime the formation of enhanced NLRP3 inflammasome which aggravate neurodegenerative disease, metabolic disease and cancer [49, 50]. Furthermore suppression of the NLRP3 inflammasome avert cardiac aging and increases lifespan in mice [51]. IL-18 is a member of the IL-1 cytokine family which is a pro-inflammatory molecule that increases with age and associated with co-morbidities [52]. *Interleukin 1 beta* (IL-1β) is another pro-inflammatory cytokine and member of the IL-1 cytokine family that has been associated with orchestration of age-associated inflammation [53]. Similarly, other biomarkers like CRP and IL-15 are also reported to increase with age [54–56].

Alongside with the appearance of pro-inflammatory cytokines, there is a concomitant increase in an anti-inflammatory molecule with age. This indicates the effort made by the immune system to maintain homeostasis. However, this aspect is often disregarded in studies on inflammation and aging. One of the anti-inflammatory molecules that increase with age is IL-10 that inhibits the action of IL-18, TNFα, and IL-6 [46]. There are varying reports on the beneficial role of having a high level of IL-10. On one hand, increased level of IL-10 has been implicated with a risk of coronary heart disease [57]. whereas, vascular dysfunctionality was improved by administering exogenous IL-10 in mice [58, 59]. The level of Transforming growth factor beta (TGF-β), another anti-inflammatory molecule, also has been reported to increase with age [60]. TGF-β has important regulatory role in old age associated pathologies like osteoarthritis. Where lack of TGF-β responsiveness in old mice result in the insufficient repair of cartilage resulted from pathogenesis of osteoarthritis [61]. TGF-β has a pleiotropic cellular functions, including cellular proliferation, migration, DNA damage repair, telomere regulation. Deregulation of TGF-β signaling has been linked with cellular senescence and age related disease like, Alzheimer’s disease (AD), osteoarthritis, and cardiovascular disease [60].

Overall, inflammaging appears both in successful aging (aging without comorbidities) and unsuccessful aging (aging with a comorbidities), perhaps, the difference could be timing as the elevation of pro-inflammatory molecules like IL-6 occur later in life in case of successful aging whereas for unsuccessful aging, they appear earlier [41]. Long-lived people, centenarians, with high IL-6 levels are able to cope with chronic inflammation through anti-inflammatory response which is called “anti-inflammaging” [62].

### Sources of inflammaging in the elderly

Different sources have been implicated for the appearance of inflammaging with age. The accumulation of damaged cells and release of macromolecules like adenosine triphosphate (ATP), fatty acids, and advanced glycation end-products (AGE) could continuously activate the innate immune system to release different cytokines [63]. The other source of inflammaging could be leak of microbiota products from the gut (change in gut permeability) and oral environment entering into the circulation [64]. The accumulation of senescence cells with age is also regarded as one source of inflammaging. Cellular senescence occurs via continuous replication of cells or it could be a result of cellular response to stress and damage. It is important in the prevention of malignancy by driving cells to a state of rest by avoiding replication. On the other hand, accumulation of senescent cells is one of the drivers of aging and possibly age-associated diseases via the release of secretory phenotypes. The senescence-associated secretory phenotype (SASP) is a profile of collective molecules including pro-inflammatory cytokines that are generated from senescence cells. SASP is able to affect the surrounding tissue microenvironment and modify the function of cells [65]. Immunosenescence is another source of inflammaging where there is a mild activation of the immune system due to an increasing burden of antigen exposure. Especially, chronic infections like cytomegalovirus (CMV) aggravates immunosenescence and inflammaging by a continuous provocation of the immune system [66].

### Inflammaging is linked with age-related diseases

Inflammaging has been associated with different age-related diseases. Pro-inflammatory cytokines like IL-6, TNFα, and C-reactive protein (CRP) have been associated with pathological conditions like Alzheimer’s disease, and cardiovascular diseases. An imbalance between pro-inflammatory lipid mediators and pro-resolving lipid mediators result in delayed resolution of inflammation resulting in formation of atherosclerosis plaque [67]. Increased level of IL-6 was also strongly associated with sarcopenia [68] and frailty [69].. Similarly, a higher level of CRP and IL-6 were also associated with self reported major mobility disability in older adults [70]. The incidence of cancer and cancer-related death was also associated with a high level of circulating IL-6, TNFα, and CRP in a five-year follow-up of elderly individuals [71]. The pathogenesis of Alzheimer’s disease is also linked with different cytokines, and inflammatory mediators [72].

Inflammatory molecules were also associated with frailty. The level of circulating IL-2Rα, sgp130, MCP-1, I-309 IL-6R, leptin, B cell-attracting chemokine 1 (*BCA-1*) and RANTES were able to predict frailty in old individuals [73]. Likewise, low cognitive performance was also associated with high level of soluble tumor necrosis factor receptor 2 (sTNFR2), sIL-2Rα, and sgp130 in elderly individuals [74]. Inflammaging is a common denominator to most age related diseases including hypertension, lung disease, obesity, diabetes, and cancer where these co-morbidities are reported to increase the risk of patients to develop severe COVID-19. Furthermore, the mortality of COVID-19 patients with co-morbidities is much higher compared to patients without co-morbidities [75, 76], which indicate the need for further scrutiny on the role of inflammaging in COVID-19 related mortality.

### Inflammaging aggravate COVID-19 disease in elderly

The spectrum of clinical manifestation among COVID-19 subjects varies from 80% of individuals having asymptomatic, mild or moderate symptoms to 20% who develop a serious disease and one-fourth progress to suffer critical respiratory failure requiring intubation and ventilator support [77]. Patients experiencing ARDS needing ventilator support are characterized by magnified inflammation. This leads to damage of alveoli wall resulting in capillary leakage of protein-containing fluid that fills up the alveoli with an outcome of respiratory failure [78]. The involvement of the immune system is evident in COVID-19 patients; significant increment of chemokines and cytokines including IL7, IL8, IL9, IL10, IL1-β, IL1RA, GM-CSF, MCP1, MIP1α, and MIP1β is observed. Severe COVID-19 patients admitted to intensive care unit showed higher level of pro-inflammatory cytokine including MCP1, MIP1α, IL2, IL7 and TNFα [79].

For an instant, IL-6, which is one of the proinflammatory molecules in the state of inflammaging in elderly individuals, is also highly correlated with mortality rate among COVID-19 patients [80]. One evidence is that corticosteroid therapy in COVID-19 patients targeting IL6 and other cytokines showed better outcome. A randomised, multicentre study pointed out the use of dexamethasone among hospitalised COVID-19 patients requiring respiratory support has significantly decreased the 28-day mortality rate [81]. On the other hand, earlier clinical use of interferon inhalation aggravated the conditions in COVID-19 subjects [82] indicating the pivotal role of cytokines storm in disease severity.

As discussed above, chronic inflammation is a phenomenon present in healthy elderly individuals without any comorbidities and it is not uncommon in diabetes patients, obese individuals and individuals with other underlining diseases [83]. The severity and unproportional mortality due to COVID-19 also occurs in elderly subjects and individuals with underlining comorbidities. One of the major pathophysiology that cause severe disease in COVID-19 patients is a cytokine storm or cytokine release syndrome [84]. Cytokine storm can be triggered by different therapies, pathogens or disease; and it is characterized by several disorders including life-threatening systemic inflammatory syndrome, immune dysregulation, multiorgan dysfunction and failure [85].

**Fig. 1:**
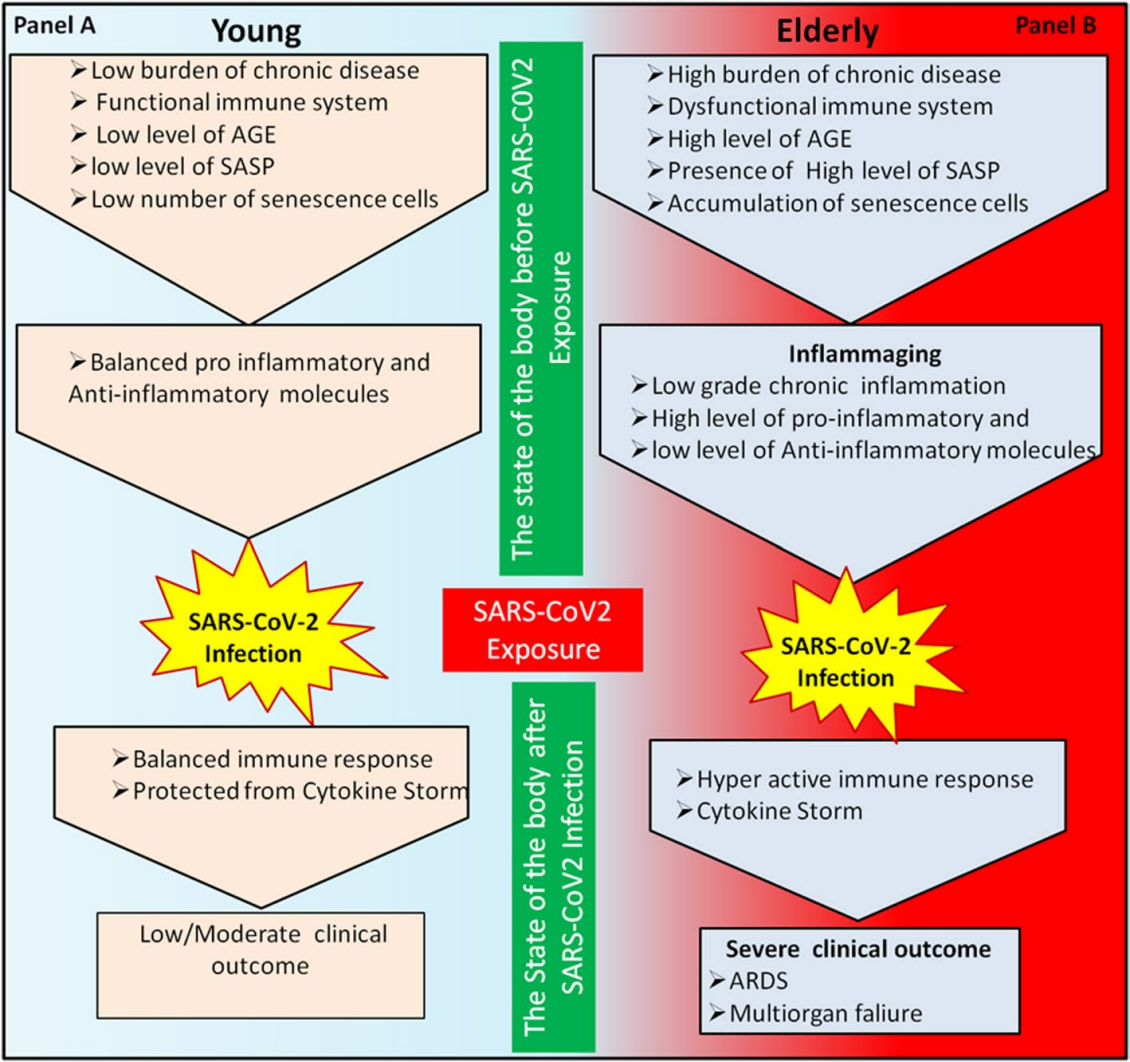
Role of inflammaging in elderly and young individuals: Young individuals have a lower burden of disease, lower level of SASP and so on which put them to have a balanced proinflammatory and anti-inflammatory molecules before SARS-CoV-2 infection. Once young individuals are exposed to the virus, the body responses in a balanced way and are protected from cytokine storm and tissue damage (Panel **A**). On the other hand, due to high burden of disease, high level of AGE, presence of high level SASP, the elderly have a more proinflammatory cytokine production when exposed to SARS-CoV-2 causing hyperactive immune response which results in cytokine storm and tissue damage (Panel **B**)

To conclude, hyper-inflammation is one of the key pathological pathways resulting in severe COVID-19 pneumonia. The presence of cytokine storm is more common in old individuals, and this could be associated with an already inflamed environment “inflammaging” in elderly aggravating the cascade of cytokine storm resulting in tissue damage leading patients to severe COVID-19 outcomes. We propose that inflammaging could play an important role in SARS-CoV-2 pathogenesis. As inflammaging is common in healthy elderly and individuals with chronic diseases, it can aggravate the host immune response to a more pro-inflammatory status leading to cytokine storm and result in tissue damage in these individuals. The role of inflammaging in elderly and young individuals is described in Fig. 1. Studies deciphering the pathways leading to cytokine storm and the role of different cytokines in disease pathogenesis are required to design better COVID-19 immunotherapeutic strategies.

### The aging immune system

The Biology of Aging is a complex process; Aging can be defined as a progressive loss of function and structure of cells, tissues, and organs resulting in impaired immune response to stress and increase vulnerability to death [86, 87]. The immune system is one of the systems that was reported as faulty with aging. The term “immunosenescence” was coined by Dr. Roy Walford (as cited by Rita B Effros) and was meant to imply an overall pathological impact of aging on the immune system [88]. Immunosenescence mainly refers to the overall reduced response to immunological challenges in the elderly group. The initial concept of immunosenescence originated from the discovery that lymphocytes stimulated from older adults and animals showed low level of proliferation and IL-2 secretion [89]. Since then, many discoveries in the field of immunology enable to understand that this is essentially due to low production of IL-2 by memory cells, which numbers increase with age, as the normal process of immunological memory.

Combating multitude of pathogens encountered throughout the human lifespan needs effective and efficient coordination of both the adaptive and innate immune system. With aging, there is a remodelling of both the innate and the adaptive immune system which can be evidenced by the low responsiveness of the elderly individuals to vaccination [90]. For instance, the global seasonal influenza associated excess mortality rate (EMR) ranged 0·1 to 6·4 per 100,000 individuals for people younger than 65 years whereas the EMR was 2·9 to 44·0 per 100,000 individuals for people aged between 65 and 74 years and EMR was much higher in elderly indivduals aged 75 and above ranging from17·9 to 223·5 per 100,000 for people [91].

Another evidence of the immune dysregulation in elderly individuals is an increased development of autoimmunity at old age [92]. A particularity of the immune system compared to the other systems is its intrinsic function: looking for stressors (pathogen) and react to it to protect the organism. As such, the immune system has been developed to specifically be stressed. In addition to acute stressors (e.g., Influenza), the immune system must deal with persistent viral infections. The contributions of both the innate and adaptive arms of the immune system are tightly regulated to fulfil these lifelong tasks.

### Cells of the innate immune system in aging and SARS-CoV-2 infection

The innate immune system acts as the first line of defense during infection. Components of the innate immune system including neutrophils, monocytes, macrophage, natural killer cells (NK cells), mast cells and dendritic cells (DCs) are involved in phagocytosis and killing of pathogens, initiating an inflammatory response, and assisting the adaptive immune response. Aging affects the number, function and the phenotype of the innate immune cells [93]. Cellular immune response plays an important role in the severity of COVID-19. Cells of the innate immune system and lymphocytes (B and T cells) of the adaptive immune systems are involved in defending against SARS-CoV-2 infection [94]. An example is that during viral infection, the immune system elicits interferons (IFNs) which in turn activate the transcription of IFN-stimulated genes (ISGs) which participate in various antiviral functions. Severe COVID-19 complication is associated with a delayed and impaired type I and type III IFN production by patients [95, 96].

### Neutrophils

Neutrophils are the first innate immune cells that are recruited to the site of tissue damage or infection. During the process of chemotaxis, neutrophils interact with the endothelial cells and extravasate to the site of infection. This interaction of neutrophils with the endothelial cell lining further activates them for consequent interaction with the microbes [97]. At the site of infection, neutrophils involve in phagocytosis, release of anti-microbial molecules (like protease, myeloperoxidase, and lactoferrin), and in the formation of neutrophil extracellular traps (NETs) to trap bacteria invasion. Once the microbes are cleared from the site of infection, they undergo apoptosis (programmed cell death) [98].

The absolute number of neutrophils between young and old individuals show no significant difference in frequency [99]. On the other hand, the functionality of neutrophils had been shown to be affected with age. Neutrophils from the elderly people show a decrease in phagocytosis activity of *Staphylococcus aureus* and *Escherichia coli* [100]. Furthermore, production of free radical to kill engulfed microbes and chemotaxis movement of neutrophils decrease with age [101].

The pathophysiology observed among severe COVID-19 patients is marked by changes in neutrophil number, phenotype, and function. Severe COVID-19 was associated with an increased number of immature neutrophils characterized with CD10^Low^CD101^−^ surface marker [102]. Early local respiratory SARS-CoV-2 infection is linked with changes in the innate system, with decreased in the number of circulating lymphocyte and increased number of neutrophils. COVID-19 patients that developed ARDS have significantly higher number of neutrophil counts compared with those without ARDS and the observed cytokine storm syndrome could also be associated with high number of neutrophils [78]. Increased activation of neutrophils and formation of neutrophil extracellular traps (NETs) is also linked with coagulopathy observed in severe COVID-19 cases [103]. Further detailed review of neutrophil response against SARS-CoV-2, see the detailed review by Reusch N et al. [104].

### Monocyte/macrophage

Monocytes and macrophages play a crucial role in fighting invading microbes and also initiate the adaptive immune system by processing and presenting antigen [105]. Macrophages are distributed throughout the body localizing in different tissues and organs and play a crucial role in homeostasis and disease. Depending on tissue microenvironment and their origin macrophage can polarize to different phenotype and function. Even if the origin of tissue-resident macrophages (TRMs) is not fully elucidated, it has been established that TRMs can arise from embryonic origin and monocyte progenitors [106]. Recent findings have showed that the M1/M2 polarization do not fully capture the different phenotypes that exist within the macrophage activation [107]. One example is circulating macrophage in patients with lung disease could have both M1 (CD80, CD86, and TLR4) and M2 surface markers (CD204, CD163, and CD206) [108].

Circulating monocytes can be differentiated into different subsets based on cell surface expression of CD14 and CD16. These subpopulations have a distinct functional role and surface receptor expression. The classical subset expresses high CD14 and no/low CD16 (CD14++CD16−/+), the intermediate subsets express CD16 and high CD14 (CD14++CD16+) while the non-classical subset expresses higher expression of CD16 with lower levels of CD14 (CD14 + CD16++) [109] There is an increased accumulation of the CD16+ pro-inflammatory monocytes with age and it was associated with a chronic inflammatory status of the elderly people [110]. The expression level of toll-like receptor 1/2 (TLR1/2) and associated signaling decrease with age. In vitro stimulation of monocytes taken from elderly individuals (> 65 years) showed a decreased production of IL-6 and TNF-α compared to young (21 years − 30 years) adults [111]. Similarly Monocytes taken from older individuals showed decreased level of TLR1 and TLR4 expression and increased level of TLR5 expression and unchanged expression of TLR2 and TLR6 when compare to monocytes from young individuals [112]. Others studies has also shown decreased TLR expression with age.

[99], decreased infiltration of macrophage to the site of infection, and a weak ability to initiate the adaptive immune response compared to young individuals [113].. Altogether, this suggests that more emphasis is needed to understand the role of monocyte and macrophages in organ-specific immunity.

SARS-CoV-2 infection leads to transcriptional and cellular changes in the upper respiratory tract and in the lung. Chemokines secreted by infected epithelial cells cause an influx of innate immune cells especially neutrophils and monocytes. In critical patients, proinflammatory macrophages were identified in the lung and could play a role in excessive inflammation and recruitment of innate immune cells [114, 115]. Patients with COVID-19 had an elevated number of monocytes [116]. Beyond the quantitative changes, phenotypic changes are also observed in patients with COVID-19. Expansion of CD14^+^CD16^+^ monocytes that express high level of IL-6 was significantly higher in COVID-19 patients admitted to ICU compared to those who did not [117]. Severe COVID-19 is associated with dysregulation of the myeloid cell compartment including the appearance of neutrophil precursors, dysfunctional mature neutrophils and HLA-DR^lo^ monocyte while CD11c^hi^HLA-DR^hi^ inflammatory monocyte is associated with mild COVID-19 [118]. Zhou et al. showed that CD14^+^ CD16^+^ expressing monocytes secret high level of IL-6 and accelerate the inflammatory process which could cause lung damage in COVID-19 patients [119]. These data indicate the presence of pro-inflammatory monocytes both in aging and in severe COVID-19 cases. For a comprehensive review of monocyte and macrophage role on COVID19, see the detailed review by Dress RJ, and Ginhoux F [120].

### Dendritic cells

Dendritic Cells (DCs) play an important role in coordinating the innate and adaptive immune system. They can be divided into two main groups: plasmacytoid DCs (pDCs) and myeloid DCs (mDCs). Furthermore, the myeloid DC can be divided into CD1c^+^ and CD141^+^ subsets [107]. pDCs are crucial during viral infection, they use TLR7 and TLR9 to recognize viral component inside the endosome and initiate an immune response by secreting both types I and type II interferon and activate NK cells for further killing of pathogens [108]. Myeloid DCs are very potent antigen presenting cells to T cells. They express different TLRs and C-type lectin which helps them recognize different pathogens [121].

Age-associated decrease in the frequency of pDCs [110], mDCs [111], and CD141^+^ mDCs has been reported [112]. The functionality of DCs is also affected with age; dysregulation of intracellular signalling like a decreased activation of the phosphoinositide 3-kinase (PI3k) in mDCs had shown to contribute for the increased amount of TNF and interleukin-6 (IL-6) in elderly individuals [113]. The functionality of pDCs is also affected with age where pDCs from elderly individuals show a reduced amount of interferon production after being stimulated with influenza virus. In addition to this, pDCs from old individuals have a reduced capacity to phagocytize and stimulate CD4 and CD8 T cells [114] suggesting dendritic cells to be affected by lifetime exposure and responses.

Severe COVID-19 is also associated with depletion in the number of pDCs and CD141^+^ (CLEC9A^+^) DCs from the blood of patients [122, 123]. Functional impairment of DCs characterized with lower expression of CD80/86 was found among patients with COVID-19 [122]. Single-cell RNA sequencing of blood APCs (antigen-presenting cells) from severe COVID-19 patients showed a deregulation of these cells compared to moderate COVID-19 patients and healthy controls. Severe COVID-19 patients were characterized by having an increased pro-apoptotic pathways in pDCs, lower level of TLR9 in pDCs, decrement in DHX36 expression in CLEC9a^+^ DCs, decreased expression of MHCII related gene in CD1c^+^ DCs and decrement of ISG in monocyte subsets [124]. Similarly Arunachalam PS et al, using single-cell transcriptomics also reported reduced HLA-DR expression in myeloid cells and lack of type I IFNs in patients with severe COVID-19 patients [125]. In general, numerical and phenotypical changes as well as dyregulation of intracellular molecules in mDCs and pDCs are observed in elderly individuals. These could be one reason why the immune system is unable to control the SARS-CoV-2 at early stage of the disease among elderly individuals leading to aggravated and complicated COVID-19 outcomes.

### Natural killer cells

Natural killer (NK) cells were first described in 1975. They are cytotoxic lymphocytes that can kill target cells without prior exposure [126]. The two main subsets of human NK cells based on the CD56 density are 1) the CD56dim NK cells which are mature and have high cytotoxic activity and 2) the CD56bright NK cells that are immature with an immunoregulatory function. Aging affects the frequency of NK cells; an increase in the number of NK cell is observed with age, the percentage of CD56bright population decrease and CD56dim subsets expand. Similarly the proliferation rate of NK cells decrease with age [127].. The CD57 expressing CD56dim population has a highly matured phenotype with increased cytotoxic activity and a reduced response to cytokines and lower proliferative capacity compared to CD57 negative CD56dim NK cells [123]. Remodelling of the NK cell subsets may contribute to the dysregulation of the rest of innate and adaptive immunity since these cells produce factors influencing the global immune response [124]. The overall granule mediated cytotoxicity of NK cell is also affected with age and the amount of IFN-γ produced by stimulated NK cells as well as per cell assay killing capacity of NK cells decrease in older adults [128].

**Fig. 2:**
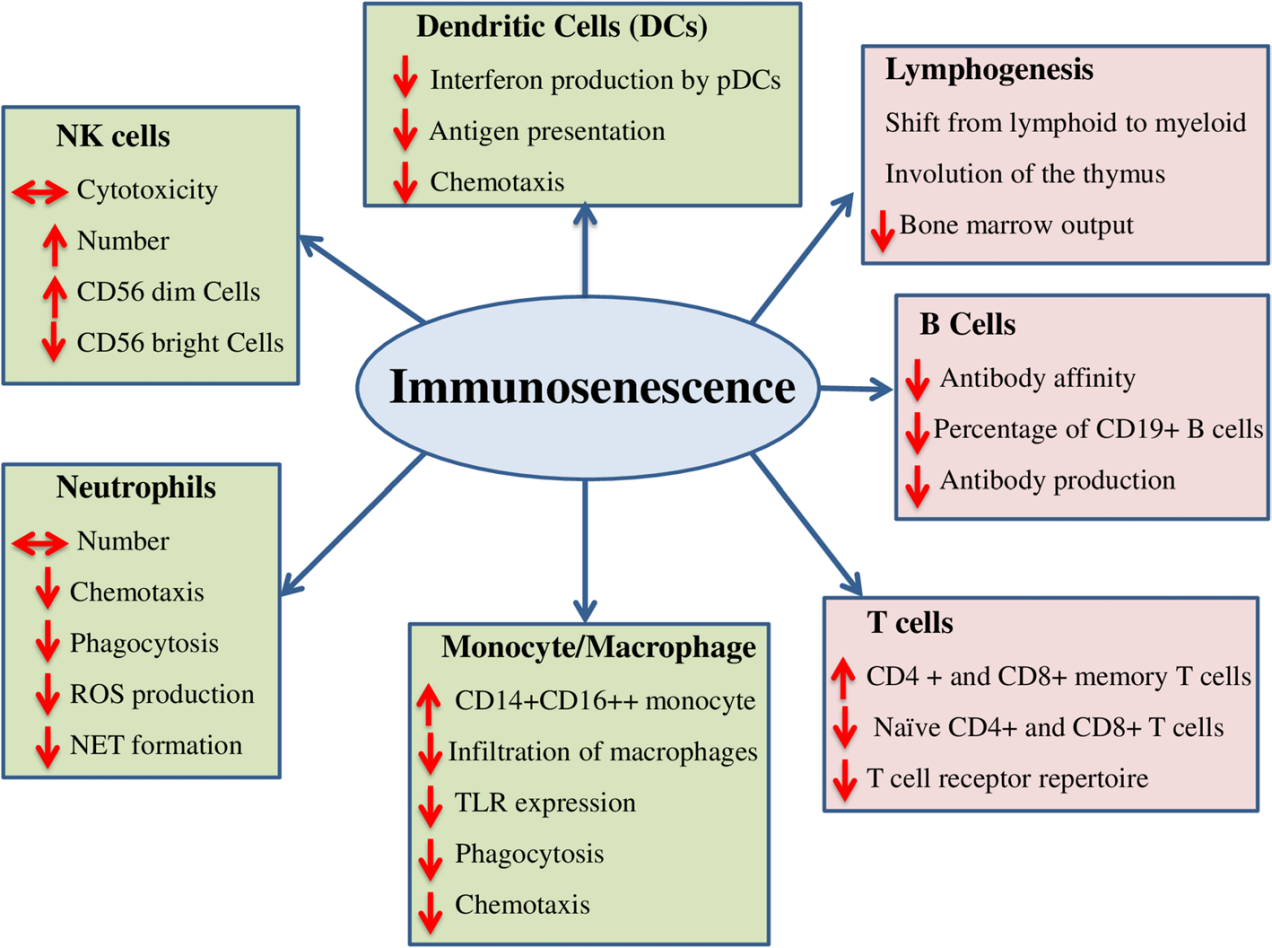
Summary of major changes associated with Immunosenescence. Aging affects the phenotype and function of different immune cells. The figure summarizes an overall impact of aging in different immune cells

NK cells are crucial in fighting viral infection, as they can directly kill infected cells without the assistance of APCs. Both phenotypical and functional changes of NK cells have been linked with different clinical outcomes in COVID-19 patients. Many have shown that severe COVID-19 cases presented significantly lower number of NK cells compared to mild cases [129]. The percentage of CD16^+^CD56^+^ NK-cells were significantly lower in patients who died of COVID-19 when compared to survivors [116]. Individuals with moderate COVID-19 had an enriched NK cell population expressing CD56^+^CD57^−^GZMK^+^ [130]. Decrement in number of NK cells and increment of NKG2A+ exhausted NK cells which produce low level of cytokines to fight infection was reported in mild and severe COVID-19 patients. An increased expression of the inhibitory marker NKG2A results in the decreased level of IFNγ, IL-2, TNFα, CD107a and granzyme B in COVID-19 patients [131]. Another study also identified other cell exhaustion markers (LAG3, PDCD1 and HAVCR2) on NK cells from COVID-19 patients which could reflect that SARS-CoV-2 infection could induce these phenotypes in COVID-19 patients [132]. Taken together, these data show that the presence of functional exhaustion in the NK cell population and remodelling of these cells with age could play some part for the innate immune system failing to control the infection at early stage. To conclude, deregulation of cells of the innate immune system in the elderly individuals allows SARS-CoV-2 to advance from early stage of infection to an aggravated disease. Fig. 2 describes age associated immunoscenesence.

### Cells of the adaptive immune system in aging and in SARS-CoV-2 infection

One of the main alterations associated with aging comes from changes observed in the adaptive immune system. The morphological changes observed in maturation organs: bone marrow for B cells and thymus for T cells indicate that dysregulation of the immune system with age may also be considered as an adaptation to the changing organism additionally to the consequences of accumulation of memory cells in the periphery due to continuous exposure to pathogens [133]. The adaptive cells are also involved in SARS-CoV-2 infection as patients are able to make antibodies and virus specific CD4 + T and CD8 + T cells. Importantly, coordinated adaptive immune response is important in controlling the disease [134]. A general decrease in the number of lymphocyte count was observed in COVID-19 patients. Compared to survivors, patients that died of COVID-19 had much lower lymphocyte count and the percentage of different lymphocyte cell subsets (like percentage of CD3+ T cells, Percentage of CD4 + T cells) was also lower [116].

### Lymphoid progenitors

Hematopoietic stem cells (HSC) are crucial in sustaining blood cell production over the entire life-span of an organism. The proper hematopoietic system entirely depends on the self-renewal and differentiation capacity of HSC. Aging results in molecular and phenotypically change in HSCs. With ageing intrinsic changes like epigenetics and chromatin architecture, proteostasis and metabolic changes detorate the HSCs potential in old age [135]. The proliferative capacity of HSCs diminish with age and a shift towards generating myeloid progenitors is observed in the elderly people. One possible explanation for the shift to myeloid lineage is an age-associated DNA methylation which results in up-regulation of genes that control the proliferation of myeloid progenitors and a down-regulation of genes that control lymphoid progenitors [136]. Studies have shown that intrinsic factors within the lymphoid-primed progenitors as the main cause for the decline in the lymphoid output. RNA seq and 5-ethynyl–2′-deoxyuridine (EdU) incorporation assay showed that increased expression of cell cycle arrest genes, lower level of Edu incorporation and proliferation in the lymphoid primed progenitors. On the other hand myeloid primed progenitors showed increased proliferation and decreased expression of cell cycle arrest genes [137]. Similarly, in old mice treated with anti-cancer drug Fluorouracil (*5*-*FU*), age-associated senescence particularly affect lymphoid progenitors whereas the myeloid progenitor growth and differentiation, bone marrow microenvironment and HSC were not affected [138]. Apart from the observed unique changes in the chromatin and epigenome organization of aged HSC, aging also alter the metabolism state of HSC, where aged HSC have a higher level of oxidative metabolism at basal state that potentially increase the ROS activity and decrease the regenerative capacity of HSC in elderly [139]. Thus understanding age associated changes in HSC is of a particular importance as these changes can pass to progeny cells and affect the whole linage of cells. Thus further scrutiny of the observed phenotypical and molecular chnges have great value in understanding haematological disorders, identifying targets for delaying the aging hematopoetic system.

### B cells

B cells can specifically identify a foreign antigen and produce an antibody to a particular antigen. The recognition via the B cell receptor (IgM/IgD) will activate naïve B cells. Activated cells further initiate the class-switching machinery (via T cell interactions) to generate specific antibody isotype (IgG/IgA/IgE). As such, B cells play an important role during vaccination. For effective antibody-mediated protection, the development of long-lived plasma cells (PCs) and high-affinity memory B cells are crucial [140].{De Silva, 2015 #127}. Phenotypically, B cells can be categorized based on the expression of CD27 as naïve and memory B cells. Naïve B cells have the expression of high levels of IgD, positive staining for IgM, and lack the expression of CD27 whereas memory B cells lack the expression of IgD and express the cell marker CD27 [141].{Agematsu, 2000 #119}.

In line with the reduced ability of HSCs to generate lymphoid progenitors, the total number of naive B cells generated inside the bone marrow decrease with age [142]. In human studies, there are different conflicting results on the subsets of B cells with age, but in most studies, they have shown a decline in the percentage of CD19+ B cells [143]. Opposing results were reported regarding the accumulation of memory B cells with age. Chong et al., found a significant decrement of CD27+ memory B cells with age [144]. whereas another study by Son et al., indicated an increase in the percentage of the memory B cell with age [145]. Others reported an increased percentage of double negative (CD19 + IgD − CD27−) memory B cells that express a lower level of HLA-DR in the elderly people [146].

The low responsiveness during vaccination associated with aging could explain functionality defect in B cells [147]. One of the essential aims during vaccination is the generation of vaccine-specific B cells and overall memory response to the target antigens and pathogens. It has been reported that a low response against pneumococcal vaccine in the elderly people was associated with a lower percentage of CD27 + IgD + IgM+ memory B cells [148]. The elderly individuals also produce a lower amount of antibody and the affinity of the antibody against the pathogen was also minimal. A decline in extrinsic factors like decline in the activity of CD4+ T cell, dendritic cell as well as changes in the B cell-intrinsic factors contribute to the decline in production of antibody in older individuals [149]. B cells ability to undergo class switch recombination (CSR) decrease with age. Similarly the expression level of (activation-induced cytidine deaminase) an enzyme needed for the initiation of CSR, and its positive regulator, the E47 transcription factor decrease with age [150]. Likewise the BCR repertoire diversity is also reduced with age, which could hinder effective response of B cells to new challenges [151].

B cells are also involved in SARS-CoV-2 infection as antibodies against the virus spike protein and Nucleocapsid is detected in COVID-19 patients. Immunoglobulin G (IgG), IgA and IgM are produced against the virus [152]. In elderly hospitalized COVID-19 patients, depletion of IgM memory B cells was correlated with superimposed infections and increased mortality [153]. Compared to individuals tested negative for SARS-CoV-2, COVID-19 patients show a specific B cell population expressing cell surface marker CD27^+^CD38^+^ [130]. Similar naïve B cell frequency was found between COVID-19 patients and healthy or recovered individuals. However, the frequency of both class-switched (IgD^−^CD27^+^) and not–class-switched (IgD^+^CD27^+^) B cells were lowered in COVID-19 patients [154]. The role of humoral response is not well understood in the aspect of COVID-19 pathogenesis. It has been shown that, from samples taken prior to the COVID-19 pandemic, around 20% individuals have SARS-CoV-2 cross-reactive antibodies which could be due to infection with seasonal human betacoronaviruses (such as OC43). These pre-pandemic antibodies do not protect individuals form infection by SARS-CoV-2 or hospitalization of COVID-19 patients [155].

Several studies have demonstrated higher antibody titers associated with a decrease in a symptomatic SARS-CoV-2 infection. Neutralizing and binding antibodies have correlated with lower risk of COVID-19 among individuals taking mRNA-1273 vaccine [156]. Six months after receiving the BNT162b2 vaccine, waning of anti-spike IgG and neutralizing antibodies was observed in elderly individuals 65 years of age or older [157]. Similarly compare to young vaccinees below the age of 60 years; elderly individuals older than 80 years old vaccinated the first and second dose of the BNT162b2 vaccine showed lower frequencies of neutralizing antibodies against SARS-CoV-2. This could implicate lower activity of B cells in this age group and further strategies should be employed to further increase vaccine response in this age group [158]. Further detailed review of B cell response against SARS-CoV-2, see the detailed review by Röltgen K, and Boyd SD [159].

### T cells

T cells are specialized lymphocytes that mature in the thymus and express uniquely rearranged T cell receptors (TCRs) used for antigen recognition. The CD4+ T cells mainly function as regulators (a subset of regulatory T cells), helper cells and recognize antigens presented on MHC-II molecule whereas the CD8+ cells have more effector functions and recognize antigens presented on MHC-I molecules [160]. The bone marrow-derived T cells migrate to the thymus to develop and become mature T cells. They undergo a series of changes starting from a double negative for CD4 and CD8 (CD4-CD8-) to double positive (CD4 + CD8+) and finally to a single positive for one of the molecules (CD4 + CD8- or CD4-CD8+) [161].

Based on their activation status T cells can be classified into naive, effector and memory subsets. The CD4 T cells can further be divided into different subsets based on cytokine production and/or expression of unique lineage defining transcription factors. These include, T helper (Th)1, that protect intracellular pathogens Th2, and Th17 which target extracellular pathogens, regulatory T cells which help maintain self-tolerance and the follicular helper T cells (T_FH_) that assist B cells for antibody production [162]. The cellular surface markers CCR7 (C-C chemokine receptor type 7) in combination with CD45RA/O, can be used to categorize the subsets of T cells. Naïve T (TN), cells are defined as CD45RA^+^CCR7^+^, central memory (TCM) as CD45RA^−^CCR7^+^, effector memory (TEM) as CD45RA^−^CCR7^−^ and effector memory re-expressing CD45RA (TEMRA) T cells as CD45RA^+^CCR7^−^ [163]. In addition to the classical T cells, there are various populations of ‘unconventional’ T cells which include γδ T cells, natural killer T (NKT) cells, and mucosal-associated invariant T (MAIT) that make up around 10% of circulating T cells. Together, these T cell subsets coordinate the immune responses which protect the host from infections and cancer [164].

In mice, the peripheral naïve T cells are maintained with a continuous supply from the thymus since the peripheral maintenance of naïve cells by division is very minimal which results in the decline of naïve CD4+ T and CD8+ T cells with age [165]. In humans, there is an involution of the thymus after puberty and most of the naïve cell pool is produced before puberty and maintained by peripheral homeostasis through proliferation [166]. Peripheral homeostatic proliferation is able to maintain the number of naïve CD4+ T cells in elderly individuals whereas the number of naïve CD8+ T cells severely reduced in old age [167]. Aging affects the function, the absolute number of circulating T cells as well as their subsets. One of the prominent futures of aging is the accumulation of memory phenotype mainly the CD8+ T subset and also in the CD4+ T cells subset [168]. T cells have the ability to recognize a myriad of pathogens using the highly diverse T cell receptor repertoire, but with age, a shrinkage of the repertoire diversity may hamper the ability to recognize newly encountered antigens [169]. The production of cytokines like IL-2 and the proliferation of naïve CD4+ T cells also decrease with age [170]. Similarly, CD8+ T cells ability to proliferate, produce cytokines and lytic proteins, lyse target cell, and up-regulate activation markers are all decreased with age [171].

Series of cellular signaling are required for the full activation, proliferation and differentiation of T cells. T cell activation is achieved by the interaction of the primary signal generated by TCR on T cell and MHC-I/II present on APCs [172, 173]. Cellular signaling generated from TCR, IL-2 receptor (IL-2R) and CD28 are altered with age. Defect in TCR signaling with age is linked with a decrease in the production of IL-2 [174]. Secondary signal produced by CD28 on T cell and B7 molecule on APCs is crucial for survival, metabolism and proliferation is also affected with age. The expression levels of CD28 decrease with age resulting in a lower proliferation capacity in these cells. The third signal comes from the interaction of IL2R with IL-2 which is important for activation of naïve cells. Alongside with the decrease production of IL-2 the activation of naïve cells decrease with age. These signaling alteration affect the functionality of the cells [175]. Besides aging, persistent virus infection such as CMV has been shown to have impact on the phenotype and functionality of T cells. CMV infection is associated with expansion of memory CD8+ T cells specific to CMV [176].

Patients infected with SARS-COV-2 were characterized with having CD4+ T and CD8+ T cells with an activated marker like HLA-DR and CD38. In severe COVID-19 patients, the exhaustion marker PD-1 was highly expressed in CD4+ and CD8+ cells [95]. The presence of SARS-CoV-2 specific CD4+ T cells was associated with effective viral clearance whereas absence of the specific CD4+ T cells against SARS-CoV-2 was linked with severe COVID-19. Loss of naïve CD4 + T cells and uncoordinated adaptive immune response in elderly individuals (> 65 years old) results in deprived disease outcome [134, 177]. Activated CD4+ T cells turned into Th1 cells expressing GM-CSF could play a role in assisting the inflammatory CD14^+^CD16^+^ monocyte to move to the lung in huge number and disable the function of the lung which leads to quick mortality [119].

CD8+ T cells pay a crucial role in fighting viral infection through direct killing of infected cells. Mild and severe COVID-19 cases were also linked with decreased number of CD8+ T cells and CD8 + T cells having an increased exhaustion marker like NKG2A [131]. COVID-19 patients showed an impaired T cells characterized with lower T cell proliferation and T cells with lower expression of TNFα and INFγ [122]. Better COVID-19 outcomes were associated with production of SARS-CoV-2 specific CD8 + T cells. In acute phase of the disease, these virus specific CD8+ T cells express high level of granzyme B, perforin and IFNγ [134, 178]. Using the expression of GZMK and GZMB, CD8^+^ T_EM_ (T Effector Memory) cells can be divided into major populations. In healthy aging process, the proportions GZMK^+^CD8^+^ T cells increase among CD8^+^ T cells and these GZMK^+^CD8^+^ T cells have been shown to contribute in inflammaging. On the other hand, COVID-19 patients show specific CD8+ T cell subsets expressing HLA-DR^+^CD38^+^PD-1^+^. Similarly in the CD4+ T cell compartment, individuals with moderate COVID-19 patients show T_EM_ TBET^+^EOMES^+^ sub-population [130]. Mathew et al *used* 200 immune features and 50 different clinical features among COVID-19 patients and compared them with recovered and healthy individuals. Authors showed that three different immunotypes were identified; the first one was associated with disease severity characterized by robust CD4+ T cell activation and activated CD8+ T “EMRAs,” (effector memory subset that re-expresses CD45RA) hyperactivated or exhausted CD8+ T cells and Plasma B cells. The second immunotype group was also associated with disease severity and characterized by less activated CD4 + T cells, Tbet^+^ effector CD4 and CD8+ T cells and proliferating memory B cells while the third group was inversely correlated with disease severity characterized by lack of activated T and B cells. Mortality due to COVID-19 was occurred in all the three immunotype groups [154]. For comprehensive review on T cell response against SARS-CoV-2, see the detailed review by Paul Moss [179].

**Fig. 3:**
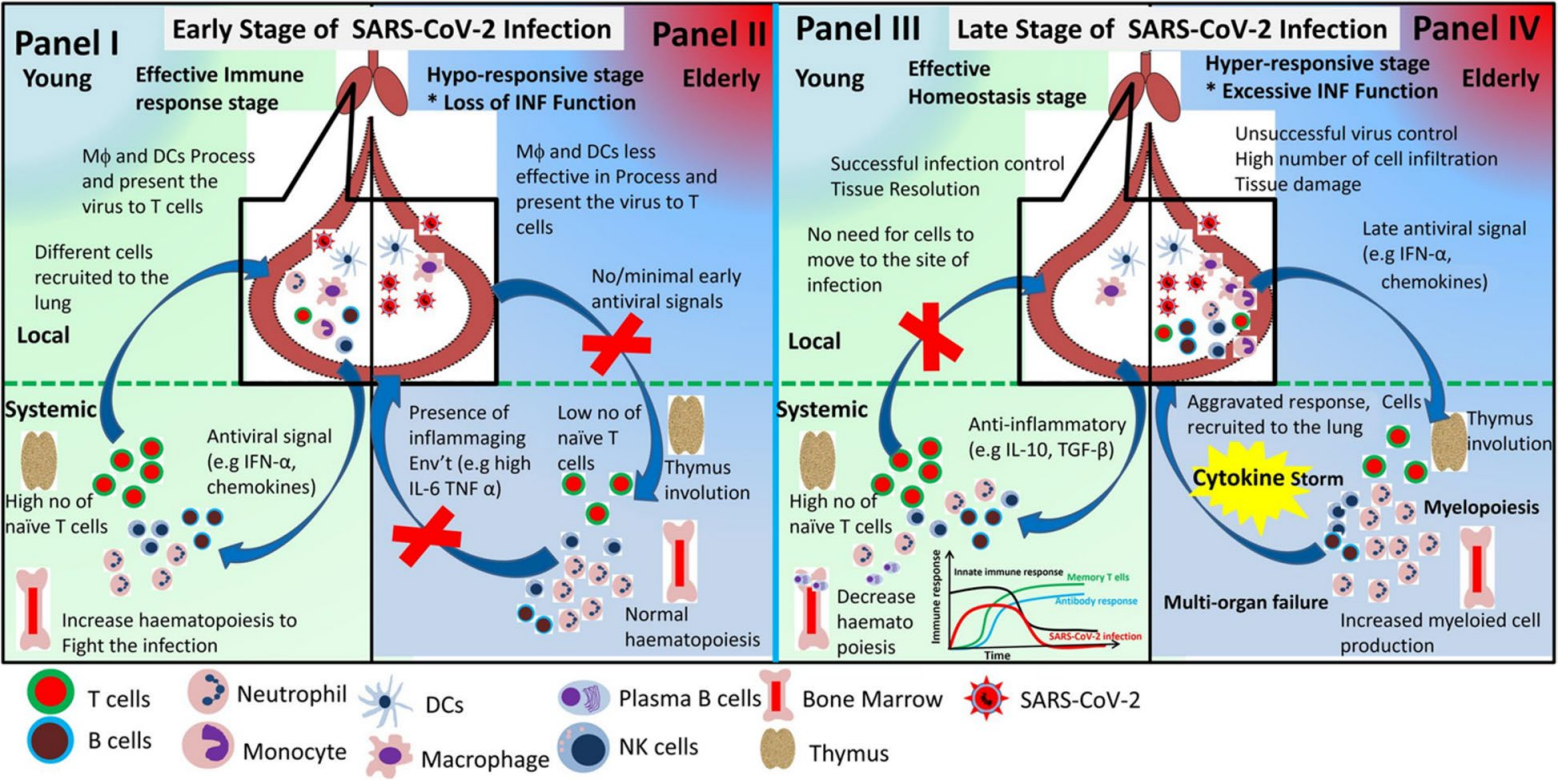
Early and late stage immune response to SARS-CoV-2 infection in young and elderly Individuals. During early stage of SARS-CoV-2 infection in the respiratory tract of young individuals the pathogen is easily recognized and different chemokines and cytokines are secreted which enables the recruitment of immune cells to the site of infection (Upper Panel I). These processes also increase hematopoesis as well as since young individuals have a large pool of naive T cells they can easily respond to new pathogens (Lower Panel I). In elderly individuals early stage of SARS-CoV-2 infection is associated with hypo-responsiveness, where the immune cells are not efficiently recruited to the site of infection (Upper Panel II), thymus involution in elderly leads to lower number of naïve T cells which minimize the control of novel pathogens (Lower Panel II). Late stage of SARS-CoV-2 infection in the young is characterized by controlling and eliminating the virus, tissue repair (Lower Panel III), blocking of cell recruitment to the site of infection, developing memory T cell, and developing anti- SARS-CoV-2 antibody (Lower Panel III). Whereas the late stage of SARS-CoV-2 infection in the elderly is characterized by hyper- responsiveness, where large number of immune cells are recruited to the site of infection (Lower Panel IV), the bone marrow turned to myelopoiesis (producing more myeloid cells) and excessive release of pro-inflammatory cytokines (especially IL-6) leads to cytokine storm which could lead to severe COVID-19 (which could include multi-organ failure) and death (Lower Panel IV)

To sum up, the adaptive immune system is key in fighting, controlling, and producing immunological memory to potential pathogens. When considering the novelty of SARS-CoV-2 to the immune system, the involution of the thymus (producing low number of naïve T cells) and the shrinkage of the T cell receptor repertoire with age make the elderly individuals unable to fight and clear the virus at early stage of the infection. The bone marrow also tends to produce more myeloid cells and the number of naïve B cell decrease plus to that the functionality of the B cells is also affected with age making the elderly to experience severe COVID-19 disease course. Fig. 3 depicts the immune response of young and elderly to early and late stage of COVID-19 course.

## Conclusion and future perspectives

The physiopathology of COVID-19 involves a complex host-virus interaction involving different immune cells and inflammatory molecules. Unbalanced immune response like hypo-responsiveness (uncontrolled viral replication) and hyper-responsiveness (disproportionate inflammation) contribute to severe COVID-19. Detailed understanding of the role of various immune cells in SARS-CoV-2 infection would be an important step for developing therapeutics. As the elderly are disproportionally affected by severe COVID-19, better understanding of the changes in the immune system with age would give important clue in the pathogenesis of the disease. Furthermore, the similarity and difference of cellular and secretory immune responses to SARS-CoV-2 infection in relation to aging could give an important insight for an immediate response to any future new pathogen.

The inflammatory environment and the immune landscape between young and old as well as within the old population are diverse. Therefore, especial care should be given when comparing and interpreting the immune response between young and old individuals. For instance, stratification of old individuals based on comorbidities, metabolic syndrome and use of drugs like metformin has reviled different inflammatory status within a controlled age group of elderly individuals [83]. Similarly, a decrease in naïve CD8+ T cells population is linked more with healthy aging rather than COVID-19 specific immune response in the elderly [130]. Some studies have shown the presence of SARS-CoV-2-reactive CD4^+^ T cells in 40–60% of previously unexposed individuals due to cross-reactivity between the common cold coronaviruses and SARS-CoV-2 [180]. Even if most individuals infected with SARS-CoV-2 develop neutralizing antibody, some patients were able to recover from COVID-19 without the help of neutralizing antibodies as they don’t develop significant amount of antibody titter against the virus [181]. On the other hand as many of the current studies compare individuals with sever COVID-19 case with less sever COVID-19 case the observed higher level of inflammation in sever COVID-19 patients could also be the result of the sever disease itself. Thus, further investigation is needed to identify the actually role of preexisting high level of inflammatory molecule on the prognosis of COVID-19. In overall these studies indicate that a careful analysis and interpretation is needed to fully unravel the immunopathology behind COVID-19.

Beside the use of vaccine as a strategy against SARS-CoV-2, anti-aging drugs have shown to have effect on the immune response in elderly individuals. In a clinical trial where elderly individuals taking low dose mTOR inhibitor for 6 month have showed an increased response to flu vaccine compared to those that take placebo [182]. Another anti-aging drug that has been tested for some time is the type 2 diabetes drug metformin. A retrospective study compared the outcome of in-hospital mortality rate among metformin users and nonusers in hospitalized COVID-19 patients with diabetes. Among hospitalized diabetes patients with COVID-19, the in-hospital mortality rate in those taking metformin was 2.9% compared to 12.3% in those who don’t take the drug [183]. Similarly senolytic drugs like Azithromycin and Quercetin, have shown to have anti-vairal activity and has been proposed for the treatment and prevention of COVID-19 infection [184]. These findings could suggest that elderly individuals taking anti-aging drugs may perhaps have unique inflammatory and immunological features that could protect them from sever COVID-19.

In this review we tried to highlight major changes in the inflammatory conditions and deregulation of the immune cells in number, phenotype and function in relation to age as well as the observed changes during SARS-CoV-2 infection. Here we hypothesized that inflammaging and immunosenescence could play an important role in SARS-CoV-2 pathogenesis and unfavourable COVID-19 clinical outcomes in elderly individuals. As inflammaging is present in healthy elderly and individuals with chronic disease, it can aggravate the host immune response to a more pro-inflammatory status leading to cytokine storm and result in tissue damage in these individuals. On the other hand, immunosenescence decrease the ability of the innate immune cells to clear or control the virus at its early stage of infection as well as to process and present it to T cells. Similarly, the adaptive immune cells are shrinked with low number of naïve cells and less able to fight novel pathogens like SARS-CoV-2. Severe clinical outcomes in elderly individuals could be linked with the changes observed in the immune system during healthy and unhealthy aging.

Therefore we recommend more research for a better understanding of the complex role of the immune response and its protective and pathogenic effects in COVID-19. Studies using age matched as well as health status matched controls would highlight why some elderly escape from severe COVID-19 while others suffer and die. Moreover, important information could be gathered for viral pathogenesis, therapeutics, vaccine response and clinical outcomes in elderly COVID-19 patients.

## Data Availability

Not Applicable.

## Abbreviations

ACE2: Angiotensin-Converting Enzyme 2
AGE: Advanced Glycation End-products
ARDS: Acute Respiratory Distress Syndrome
COVID-19: Coronavirus Disease 2019 (COVID-19)
CRP: C-reactive protein
DCs: Dendritic Cells
HCoV: Human Coronavirus
IG: Immunoglobulin
IL: Interleukin
NK cells: Natural killer Cells
RAAS: Renin-Angiotensin-Aldosterone-System
SARS-CoV-2: Acute Respiratory Syndrome Coronavirus 2
SASP: Senescence-Associated Secretory Phenotype
TEM: T Effector Memory
TGF-β1: Transforming Growth Factor-β1
TMPRSS2: Transmembrane Serine Protease 2
TNFα: Tumor Necrosis Factor alpha
